# Interfacial Engineering Strategy for High-Performance Zn Metal Anodes

**DOI:** 10.1007/s40820-021-00764-7

**Published:** 2021-12-02

**Authors:** Bin Li, Xiaotan Zhang, Tingting Wang, Zhangxing He, Bingan Lu, Shuquan Liang, Jiang Zhou

**Affiliations:** 1 School of Chemical Engineering, North China University of Science and Technology, Tangshan, 063009 People’s Republic of China; 2 School of Materials Science and Engineering, Key Laboratory of Electronic Packaging and Advanced Functional Materials of Hunan Province, Central South University, Changsha, 410083 People’s Republic of China; 3 School of Physics and Electronics, Hunan University, Changsha, 410082 People’s Republic of China

**Keywords:** Interfacial engineering, Zn anode, Dendrites, Side reactions, Aqueous zinc-ion batteries

## Abstract

Due to their high safety and low cost, rechargeable aqueous Zn-ion batteries (RAZIBs) have been receiving increased attention and are expected to be the next generation of energy storage systems. However, metal Zn anodes exhibit a limited-service life and inferior reversibility owing to the issues of Zn dendrites and side reactions, which severely hinder the further development of RAZIBs. Researchers have attempted to design high-performance Zn anodes by interfacial engineering, including surface modification and the addition of electrolyte additives, to stabilize Zn anodes. The purpose is to achieve uniform Zn nucleation and flat Zn deposition by regulating the deposition behavior of Zn ions, which effectively improves the cycling stability of the Zn anode. This review comprehensively summarizes the reaction mechanisms of interfacial modification for inhibiting the growth of Zn dendrites and the occurrence of side reactions. In addition, the research progress of interfacial engineering strategies for RAZIBs is summarized and classified. Finally, prospects and suggestions are provided for the design of highly reversible Zn anodes.

## Highlights

- The interfacial engineering strategies of surface and electrolyte modifications for high-performance Zn metal anodes are reviewed.
- The reaction mechanisms for inhibiting dendrite growth and side reactions in interface engineering are systematically summarized.
- An outlook on future reference directions for new interface strategies to advance this field is provided.

## Graphical abstract

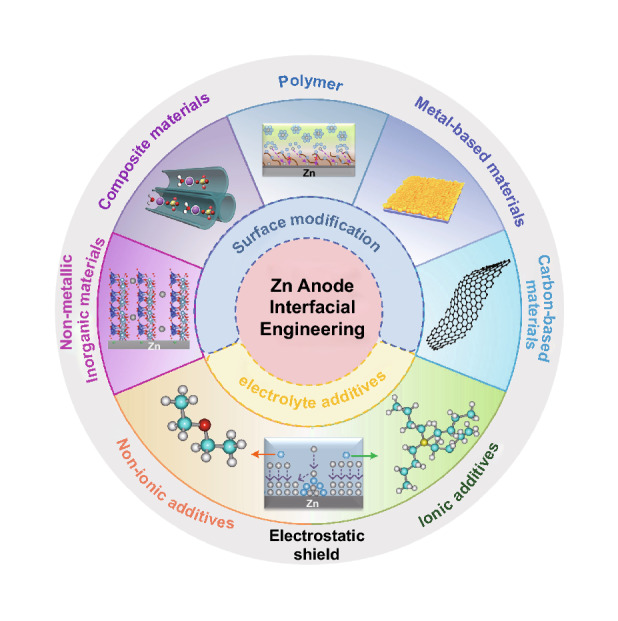

## Introduction

The two major issues of energy and environment are closely related to social development and human survival. With the depletion of fossil resources, such as petroleum and coal, and the threat of an increasingly deteriorating environment, the development of renewable energy sources, such as hydropower, solar energy, and wind energy, has become a global trend [1–4]. As an efficient electrochemical energy storage-conversion device, batteries can integrate the unstable energy obtained and output it into the smart grid for use [5, 6]. Secondary batteries are a good choice, and their commercialization has provided great convenience to society [7, 8]. Lithium-ion batteries currently dominate the commercial market, but their high-cost and safety issues, arising from the use of organic electrolytes, hinder their further development [9–14]. For example, the battery pack of a Boeing 787 aircraft ignited in 2013, a Samsung Note 7 mobile phone exploded in 2016, and a Tesla Model S electric car battery spontaneously ignited in 2019, all of which were caused by the flammability of organic electrolytes. Therefore, the security issue is a non-negligible problem that must be resolved. Coupled with the urgent need for resources and environmental protection, researchers are urged to vigorously explore new battery systems with high safety, high-cost performance, green environmental protection, and high specific capacity [15, 16]. Rechargeable aqueous Zn-ion batteries (RAZIBs) have become one of the best choices for large-scale energy storage systems because of their high safety, high capacity, low cost, and low redox potential [17–23].

The Zn-MnO_2_ battery using an alkaline electrolyte, which can be charged and discharged repeatedly, was successfully developed in the 1960s. The energy storage mechanism of alkaline Zn batteries is mainly that of a conversion reaction. Moreover, in alkaline Zn batteries, positive and negative electrodes are prone to irreversible side reactions, resulting in low Coulombic efficiency (CE) and poor cycle performance [24]. In 1988, Shoji et al. [25] took the lead in using a weakly acidic electrolyte (ZnSO_4_) instead of an alkaline electrolyte to design a new aqueous rechargeable Zn-MnO_2_ battery. Unlike alkaline Zn-Mn batteries, some by-products, such as ZnO and Zn(OH)_2_, are generated on metallic Zn. New aqueous Zn-based secondary batteries that use Zn^2+^ as charge carriers in aqueous electrolytes have recently received increasing attention for large-scale energy storage applications [26–28]. Redox reactions involving multiple electron transfers provide higher energy densities [29]. The ionic conductivity of aqueous electrolytes is two orders of magnitude higher than that of organic electrolytes [30].

**Fig. 1:**
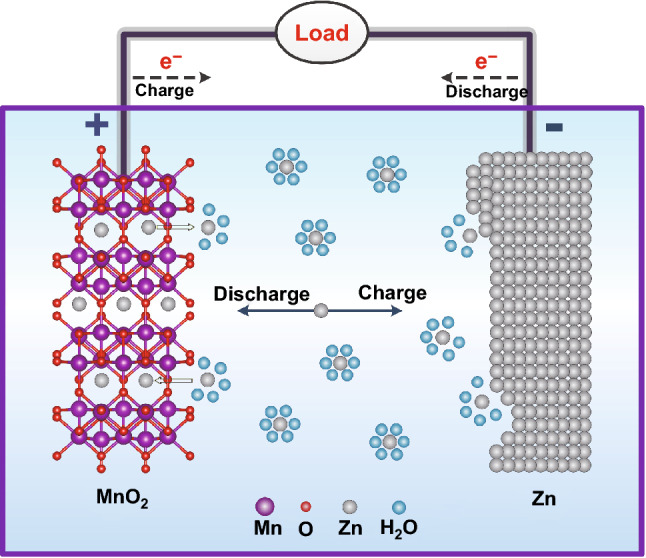
Schematic diagram of the working mechanism of RAZIBs

During the charging process, Zn ions are extracted from the cathode and are plated on the Zn anode. During the discharging process, Zn ions are stripped from the anode and inserted into the cathode material, as shown in Fig. 1. Typical cathode electrode materials for RAZIBs, including Mn- and V-based compounds, Prussian blue analogs, organic compounds, and polysulfide, usually have a tunnel or layered structure which allow the insertion and extraction of Zn ions and provide storage sites for Zn ions [31–35]. There are usually two types of negative electrodes according to the different reaction mechanisms, namely the insertion/extraction type (Na_0.14_TiS_2_ [36], Mo_6_S_8_ [37], and ZnMo_6_S_8_ [38]) and the plating/stripping type (metallic Zn [39]). The theoretical capacity of metallic Zn is 820 mAh g^−1^, which is higher than that of intercalated-type anode materials [40, 41]. In aqueous electrolyte, Zn features the unique characteristics of a low redox potential (− 0.76 V vs. standard hydrogen electrode (SHE)) and a high hydrogen evolution overpotential (~ 1.2 V vs. SHE) [42, 43]. In addition, Zn exists as a trace element in the human body, and Zn compounds are also environmentally friendly. Metal Zn is abundant in the earth's crust, which helps to reduce production costs [44]. Based on the above characteristics, metallic Zn foil (ZF) is a promising and common anode material for aqueous Zn-ion secondary batteries [45, 46].

When pure metal ZF is directly used as the anode in RAZIBs, the Zn^2+^ plating/stripping process occurs continuously on the surface of the Zn electrode during charge–discharge cycling, which dominates the reversibility of the RAZIBs [47]. The current problems with Zn anodes are primarily Zn dendrites and side reactions [48, 49]. Owing to their lower surface energy and higher migration energy, Zn ions exhibit a propensity to deposit on a lumped site to form dendrites rather than toward nearby areas. The depositions continue to accumulate and form dendrites, which can pierce the battery separator and even cause short circuits [50, 51]. This will adversely affect the battery performance, including capacity, CE, and cycle life. Moreover, Zn metal anodes inevitably encounter side reactions related to water molecules, such as corrosion and hydrogen evolution reactions. Inert by-products (ZnO, Zn(OH)_2_, etc.) caused by side reactions will increase the surface roughness of the Zn anode and decrease the number of active sites for Zn-ion deposition, resulting in an uneven distribution of electrolyte flux, electrode polarization, and capacity degradation [52, 53]. The side reaction of hydrogen evolution and the plating/stripping reaction of Zn are competing reactions, which will reduce the utilization rate of the Zn anode. However, the problems in Zn metal anodes are not isolated but interactional. Zinc dendrites can not only penetrate the separator to induce battery failure but can also provide increased surface area to exacerbate side reactions on the Zn anode surface. The side reactions increase the surface roughness of the Zn anode and provide more nucleation sites for Zn ions, which aggravates the growth of Zn dendrites. Therefore, to comprehensively solve these Zn metal anode problems, it is necessary to consider the influence of multiple problems at the same time.

Both Zn dendrites and side reactions are closely related to the Zn anode interface toward the electrolyte. Interfacial engineering, including surface coating and the addition of electrolyte additives, is an effective strategy to regulate the deposition behavior of Zn ions and the effect of water molecules, which effectively alleviate Zn dendrite growth and the Zn anode side reactions [54–57]. Surface coatings can reduce direct contact between the Zn anode and water molecules in the electrolyte, inhibiting side reactions on the Zn anode surface. Furthermore, the addition of ions or organic additives to the electrolyte can induce uniform deposition of Zn ions. The anode interfacial engineering strategy of RAZIBs has the advantages of simplicity and high efficiency and is expected to be applied to large-scale energy storage systems. This review mainly focuses on the intrinsic mechanism of interfacial engineering and the modified material types of metal Zn anodes, with the aim of providing guidance for the development of high-performance Zn-ion batteries. Finally, the future development prospects and directions of the interfacial modification of Zn anodes are presented. It is hoped that researchers will explore high-performance and long-life RAZIBs to meet the needs of large-scale energy storage systems.

## Protection Mechanism of Interfacial Engineering

The elimination of Zn dendrites and suppression of side reactions have attracted widespread attention. Regulating the deposition behavior of Zn ions by surface coating protection and electrolyte modification is an effective strategy to obtain a high reversibility and utilization rate of Zn anodes. However, there are different protection mechanisms. The following is a detailed summary of the two protection mechanisms of interfacial engineering for inhibiting dendrite growth and side reactions.

### Inhibiting Dendrite Growth

**Fig. 2:**
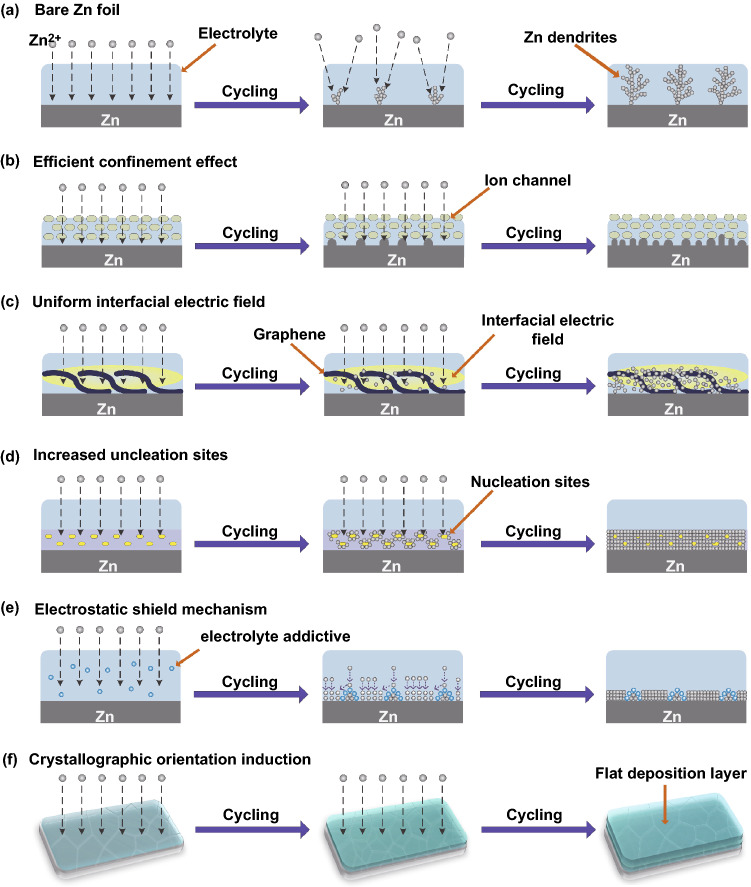
**a** Schematic diagram of Zn dendrite formation on bare Zn. Schematic diagram for various dendrite suppression mechanisms on the coating layer. **b** Efficient confinement effect. **c** Uniform interfacial electric field. **d** Increased nucleation sites. **e** Electrostatic shield. **f** Crystallographic orientation induction

In the charging and discharging process, Zn ions in electrolyte deposit and dissolve repeatedly on the anode surface. The uneven distribution of the electric field on the anode surface and the unrestricted two-dimensional (2D) diffusion of Zn^2+^ absorbed on the anode surface lead to the formation of Zn dendrites [58, 59] (Fig. 2a). Specifically, Zn ions tend to deposit at charge transfer sites on the anode with favorable energy, forming initial tiny bumps. To reduce the surface energy, subsequent Zn ions tend to deposit at these bumps, which makes them grow gradually and become primary dendrites. Under normal conditions, brittle dendrites are needle shaped; their tips act as charge centers in subsequent reactions and trigger tip effects, which further intensifies the uneven distribution of the electric field on the anode surface and causes dendrite evolution [60]. When the Zn dendrite grows to a certain extent, it punctures the separator and causes the battery to short circuit, which severely affects the battery capacity and cycle life [61]. Brittle dendrites that break off from the anode surface result in the production of "dead zinc," accelerating the depletion of Zn, which is harmful to electrochemical behavior [62].

Coating a protective layer on the anode surface can create a physical protection layer between the metal Zn anode and electrolyte, which is an effective method for improving the Zn electrode interface stability and cycle endurance. The main purpose of constructing an artificial interface layer is to regulate the deposition behavior of Zn ions and suppress Zn dendrites. Moreover, the protective layer can prevent direct contact of the Zn anode with the electrolyte, avoiding side reactions [62]. The electrolyte additive that modifies the Zn anode interface is adsorbed on the surface of the Zn anode, forming an electrostatic shielding layer. Thus, Zn ions are deposited downward instead of at the tip [63]. Based on investigations of the employed coating materials, four reaction mechanisms for inhibiting dendrite growth on the anode interface, including efficient confinement effect, uniform interfacial electric field, increased nucleation sites, and electrostatic shielding, are summarized.

#### Efficient Confinement Effect

Some inorganic oxides with high dielectric constant and low conductivity have efficient confinement effects on the electrodeposition behavior of Zn ions. Inorganic oxide coatings with porous channels could restrict the migration path of Zn ions and drive Zn ions to orderly deposit from bottom to top along the channels rather than randomly (Fig. 2b). Similarly, some inorganic salt coatings induce the uniform deposition of Zn ions, which contributes to the redistribution of Zn ions and reduces the ion concentration gradient generated by preferential ion flux near the Zn nucleation clusters [64]. In addition, this type of inorganic coating shapes the uniform flux of the electrolyte through confinement effects and further guides the Zn plating rate on the Zn electrode surface. The limiting effect of the nonporous structure and the large potential change between the electrically insulated coating-medium prevents the formation of dendrites.

#### Uniform Interfacial Electric Field

The electrochemical reaction on the Zn anode surface is closely related to the plating and stripping of Zn ions. In general, Zn-ion deposition is driven by the electric field, and the intensity and uniformity of the electric field directly affect the nucleation and deposition of Zn ions. Therefore, a uniform interfacial electric field is of great significance for compact and plain nucleation of Zn ions and improvement of cyclic stability. It should be noted that during nucleation, the disordered deposition of Zn ions on the anode surface considerably affects the relatively uniform electric field distribution [65]. Specifically, Zn-ion concentration is low in areas with low electric field intensity and high in areas with high electric field intensity. With the rapid deposition of Zn ions, the nucleation size increases and the concentration of Zn ions increases consecutively. Coating materials with high conductivity, such as carbon-based and metal materials, provide a strong electric field strength and accelerate the transfer rate on the anode surface. In this case, a highly conductive layer with a stable electric field effectively restrains the accumulation of electric charge and provides a uniform surface electric field, inducing a uniform Zn deposition behavior and avoiding dendrite proliferation (Fig. 2c).

#### Increased Nucleation Sites

Zinc ion deposition initially tends to occur at favorable nucleation locations with a relatively low nucleation overpotential [66]. Subsequently, the uneven deposition of Zn ions leads to dendrite growth. Balanced deposition of Zn ions, guided by artificially ample and uniform nucleation sites, can inhibit dendrite growth and obtain smooth Zn deposits (Fig. 2d). The increased number of active sites helps to reduce the overpotential of nucleation and thus refines the nucleation size, which is conducive to the uniform growth and dissolution of Zn on the surface. Some high-molecular polymer coatings exhibit zincophilicity because of their large number of polar functional groups and strong cooperation with Zn ions [64]. This provides numerous nucleation sites and helps to form a uniform deposition morphology. The strategy of introducing zincophilic sites is expected to inhibit dendrite growth. A carbon host is used as a model system, and the nitrogen site is the zincophilic site. The host with zincophilic sites exhibits uniform Zn deposition and improved electrochemical performance [67]. The construction of a three-dimensional (3D) coating interface also has a significant impact on the uniform deposition of Zn. For example, Zhou et al. [68] constructed a 3D nanoporous ZnO structure in situ on a Zn plate. The novel 3D structure provided more nucleation sites for Zn deposition and guided the ordered deposition of Zn ions through the electrostatic attraction of Zn^2+^. In addition, reduced graphene oxide (rGO) with a 3D structure provides a large electroactive area for Zn electrodeposition, and the corresponding nucleation sites increase accordingly [69].

#### Electrostatic Shield

Functional additives effectively suppress Zn dendrites, induce the uniform deposition of Zn ions, and form a flat surface. The main method is to form an electrostatic shielding layer so that Zn ions deposit uniformly downward. Ionic additives with a lower reduction potential can be preferentially adsorbed on the surface of the Zn tips to form an electrostatic shielding layer, inhibiting the growth of Zn dendrites [70]. Additionally, a positive charge repulsion exists between the cations of the electrostatic shielding layer and the Zn ions. This prevents the Zn ions from depositing on the Zn tips to form larger Zn dendrites and induces the deposition of Zn ions to form a uniform and smooth deposition layer, which is consistent with the non-ionic additive [71] (Fig. 2e). In addition, additives increase the overpotential of Zn anode deposition, which could increase the nucleation rate of Zn ions and suppress Zn dendrites. Furthermore, additives inhibit the 2D diffusion of Zn ions on the Zn anode surface to provide fewer nucleation sites, inhibiting the formation of Zn dendrites.

#### Crystallographic Orientation Induction

Crystallographic orientation induction is a promising method to adjust the electrochemical performance of Zn anodes by manipulating the crystallographic orientation of Zn deposition. Generally, the larger the angle between the growth direction of Zn dendrites and the surface of the Zn anode, the more favorable it is for the growth of dendrites. The crystal orientation affects the surface morphology and dendrite growth state by affecting the direction of crystal growth [63]. The angle between the crystal growth direction and the substrate, which can effectively alleviate the growth of Zn dendrites and occurrence of corrosion reactions, resulting in smooth deposition, is 0°–30° [72]. The angle between the crystal growth direction and the substrate is 70°–90°, which is very conducive to the growth of dendrites. Therefore, the specific crystallographic structure exposed in parallel with the current collector can induce the deposition of metallic Zn, which may facilitate the stable stripping/plating of Zn in a mild electrolyte [73]. In addition, the specific crystallographic structure can induce a flat Zn deposition morphology, which reduces the contact area between the electrolyte and Zn anode during cycling and effectively suppresses the growth of dendrites and generation of by-products [74] (Fig. 2f).

### Reducing Side Reactions

**Fig. 3:**
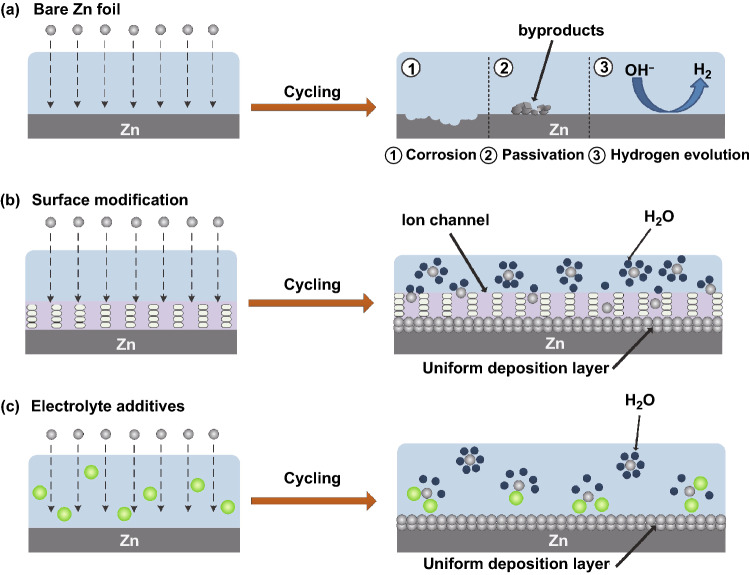
**a** Schematic diagram of the corrosion, passivation, and hydrogen evolution reactions on bare Zn. **b** Schematic diagram of the morphological evolution of coated Zn. **c** Schematic diagram of the deposition morphology of Zn ions by adding electrolyte additives

The growth of Zn dendrites increases the surface area of the Zn anode. Surface-dependent reactions, such as corrosion reactions and the hydrogen evolution reaction, cause the continuous consumption of active Zn and fundamentally reduce the battery capacity [75]. When the side reaction of hydrogen evolution occurs in a local high-energy region, the gas will cause volume expansion of the batteries. Concurrently, the local OH^−^ concentration increases and insoluble Zn(OH)_2_ is formed and adheres to the metal Zn surface, causing surface passivation of the fresh Zn [76, 77]. This reduces the conductivity of the anode, increases the interface impedance, and reduces the active nucleation sites of Zn, resulting in a poor plating/stripping CE. These irreversible hydrogen evolution, corrosion, and passivation side reactions will fundamentally consume limited electrolyte and Zn ions and endanger the performance and lifespan of batteries. As shown in Fig. 3a, a schematic diagram for Zn corrosion, passivation, and hydrogen evolution is displayed.

#### Reducing Active Water

The main reason for side reactions is that Zn^2+^ combines with six water molecules in aqueous electrolytes to form hydrated Zn^2+^ ([Zn(H_2_O)_6_]^2+^). [Zn(H_2_O)_6_]^2+^ must undergo a desolvation process before being reduced on the surface of the Zn anode, which inevitably causes direct contact between the Zn anode and water molecules and triggers side reactions. While resolving the side reaction problem, it was found that introducing atomic groups or solid electrolyte interface layers on the anode surface is beneficial for increasing the hydrogen evolution potential of metal Zn and reducing the corrosion reaction [62]. Furthermore, the interfacial layer directly prevents direct contact between the electrolyte and Zn anode or reduces the number of water molecules that reach the Zn surface through the desolvation effect [78, 79]. The Zn deposition is very uniform after the protection layer, as shown in Fig. 3b. When Zn ions are thermodynamically unstable in aqueous electrolytes, the severe passivation of the ZF by water aggravates the electrochemical behavior and reduces the CE. The artificial organic polyamide (PA) coating has rich polar groups and a cross-linked H bond network, which can combine with coordinated water molecules to destroy the solvation sheath of Zn^2+^ [80]. The novel structure coating of a 3D nanoporous Zn oxide coating accelerates the transport and deposition kinetics of Zn^2+^ by electrostatically attracting Zn^2+^ instead of [Zn(H_2_O)_6_]^2+^ [68].

#### Modulating Coordination Status

The high overpotential generated by the strong Coulomb interactions between the solvated Zn^2+^ and its surrounding H_2_O shell accelerates parasitic water reduction during Zn deposition [81, 82]. This promotes the evolution of H and the formation of a passivation layer. To inhibit water reduction and Zn dendrites, the bonding strength between the Zn^2+^ ions and solvated H_2_O should be weakened. Introducing certain additives is a simple and effective strategy for optimizing the electrolyte composition (Fig. 3c). Some additives can replace H_2_O in the Zn^2+^-solvated sheath, preferentially solvate with Zn ions, and remove H_2_O from the Zn^2+^-solvated sheath [83]. In addition, some additives also have a strong interaction with water, which reduces water activity and effectively inhibits parasitic water reactions and dendrite growth [84]. Furthermore, an in situ solid electrolyte interphase (SEI) layer can be constructed by adding additives to the electrolyte. The presence of additives not only adjusts the structure of the solvent sheath of Zn ions, allowing the rapid transmission of Zn^2+^, but also prevents H_2_O from penetrating the surface of the Zn anode, which greatly reduces the probability of side reactions [83, 84].

## Surface Modification of Zn Anode

The purpose of constructing a surface coating is to achieve uniform Zn nucleation and a flat Zn deposition layer by regulating Zn^2+^ deposition, which effectively improves the interface stability and cycle lifetime of the Zn anode. According to previous reports, various materials have been used as interfacial layers to achieve high-performance Zn anodes, carbon-based materials, metal materials, inorganic non-metals, polymers, and composite materials. The research progress of these five interfacial coating materials is described in detail below.

### Carbon-Based Materials

The key to the anode modification of RAZIBs is to reduce the growth of Zn dendrites and the occurrence of side reactions, such as hydrogen evolution and self-corrosion reactions [85]. Carbon materials have the natural advantages of high conductivity, wide range of sources, low price, environmental friendliness, and high stability. Therefore, many studies have focused on acquiring anodes with carbon-based materials with high specific capacitance [86]. Graphene, carbon nanotubes (CNTs), activated carbon (AC), and other carbon-based materials are suitable materials for modified Zn anodes. Concurrently, carbon-based materials provide abundant nucleation sites, ensure uniform Zn deposition, and avoid the generation of dendrites, which contribute to improving the electrochemical performance.

**Fig. 4:**
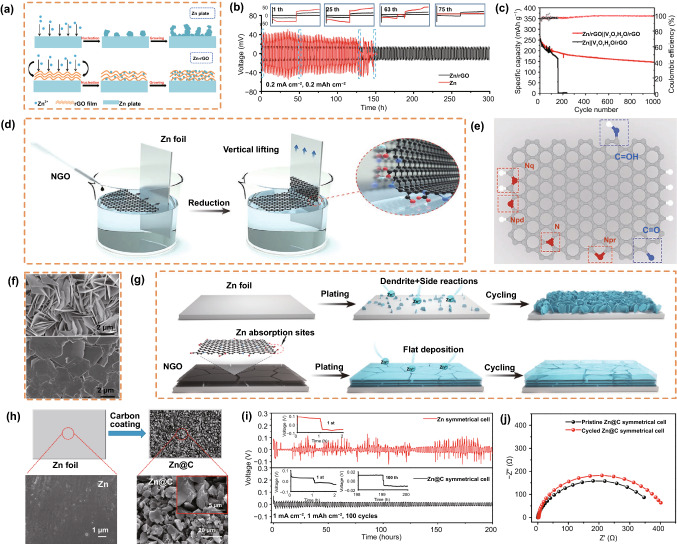
**a** Schematic diagram illustrating the Zn plating behavior of the bare Zn and Zn/rGO anodes. **b** Cycling performance of symmetrical cells using Zn/rGO and a bare Zn plate at 0.2 mA cm^−2^ with a stripping/plating capacity of 0.2 mAh cm^−2^. Copyright 2018 American Chemical Society [87]. **d** Schematic illustration of the fabrication of ultrathin graphene layers on ZF.** e **Configuration of N- and O-doped graphene. **f** Top-view SEM images of a bare ZF electrode and NGO after plating at 1 mAh cm^−2^. **g** Schematic illustration of Zn plating on a bare ZF and NGO@Zn electrode. Copyright 2021 Wiley-VCH [74]. **h** Schematic illustration of a Zn@C film and SEM images of the Zn and Zn@C. **i **Stripping/plating performance of Zn and Zn@C cells at 1 mA cm^−2^ with 1 mAh cm^−2^.** j** Nyquist plots of the Zn@C symmetrical cell before and after 100 cycles. Copyright 2018 American Chemical Society [90]

rGO has a layered structure, which provides a stable scaffold with good mechanical properties. Zinc ions can be released from the ZF through the natural ion channels between graphene layers to avoid ramification, which improves the cyclic stability of the battery. Liu et al*.* [69] studied a method for the spontaneous reduction of graphene oxide (GO) on a Zn metal surface and implemented self-assembly to form layer-after-layer structures. Layered rGO provides numerous nucleation sites for Zn nucleation, ensuring the uniform deposition of Zn (Fig. 4a). The Zn/rGO electrode showed a stable voltage curve and small hysteresis, while the ZF showed a large voltage plateau (Fig. 4b). Shen et al. [87] used Zn/rGO and V_3_O_7_·H_2_O/rGO composites as anodes and cathodes. It was found that the cell with Zn/rGO as the anode could be charged/discharged more than 1000 times at a rate of 5 C, while the cell based on bare Zn short-circuited in the 155th cycle due to dendrite growth (Fig. 4c). Compared with the Zn||Zn symmetrical cell, the Zn/rGO||Zn/rGO symmetrical cell maintained a smaller impedance after different cycles. Epitaxial electrodeposition, as a method to create highly reversible metal anodes, can be realized by textured conductive electrode coatings with a low lattice mismatch to the (002) texture of Zn metal. Zheng et al*.* [88] designed a fluid-based method to form an ordered graphene coating on a Zn surface. On the low lattice mismatch interface composed of well-arranged graphene sheets, a more uniform and compact thin plate parallel to the substrate was produced, and the newly deposited Zn layer adhered to the surface of the Zn formed in the first stage to produce a uniform metallic Zn coating. Xie et al. [74] used the Langmuir–Blodgett method to synthesize an artificial interfacial film of N-doped GO (NGO) in one step and obtained an ultrathin and parallel interfacial layer to modify the Zn anode (Fig. 4d). The parallel NGO lamellae could adjust the deposition sites of Zn^2+^ and effectively induce the deposition of metallic Zn on the (002) crystal plane. This flat deposition morphology effectively suppressed dendrite formation and side reactions. Density functional theory (DFT) was used to further study the effect of heteroatom doping of N and O on the Zn interaction (Fig. 4e). After deposition of 1 mAh cm^−2^, the bare Zn anode exhibited an anisotropic platelet-like dendritic morphology. Compared with the bare Zn electrode, even when imaged at a greater magnification, a flat and smooth surface can be obtained (Fig. 4f). The NGO@Zn electrode can achieve a flat deposition morphology and a stable interface owing to the uniform electric field and directed Zn^2+^ distribution/adsorption (Fig. 4g).

AC, a well-known carbonaceous material with good electrical conductivity and excellent chemical stability, has a complex pore structure, large specific surface area, high adsorption capacity, and high degree of surface reactivity. Xu et al. [89] prepared a Zn anode by mixing Zn powder with an AC mixture, acetylene black, polyvinylidene fluoride (PVDF), and methyl-2-pyrrolidone. The ZnAB + 12 wt% AC anode showed a relatively higher discharge plateau voltage and lower charge plateau voltage throughout the charge/discharge process. When AC is added to the Zn anode, the deposition of inactive anode products preferentially occurs in the rich pores of AC rather than on the surface of Zn particles; thus, the neatness and activity of Zn particles can be maintained even after dozens of cycles. In addition, Wang et al. [90] pressed AC on ZF (Zn@C) to create a modified Zn anode cell (Fig. 4h). After 100 cycles at 1 mA cm^−2^, plate-like Zn dendrites appeared on the surface of the ZF, yet no apparent change was observed in Zn@C. Moreover, the Zn@C symmetrical cell exhibited the stable curve of a symmetrical cell, as compared to bare Zn (Fig. 4i). Analysis of the results after the impedance test of Zn@C showed that the charge transfer resistance increased slightly (Fig. 4j). The stable stripping/plating can be attributed to the equalized charge distribution and sufficient regulation of Zn deposition after the introduction of the C layer.

**Fig. 5:**
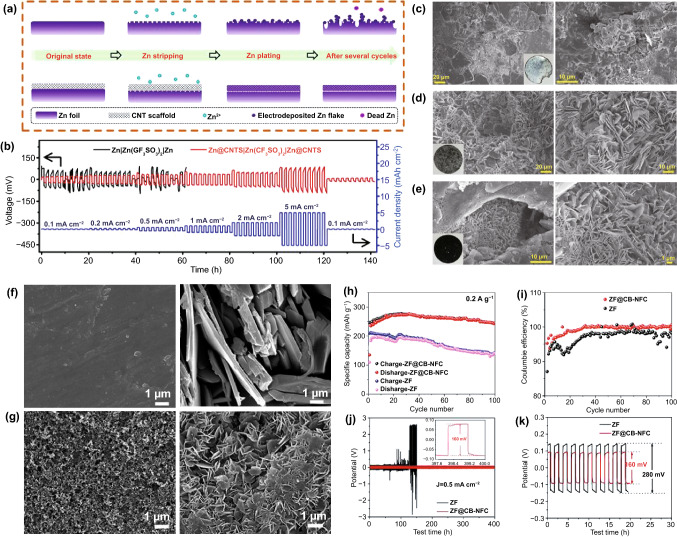
**a** Schematics of the stripping/plating behaviors of bare ZF anodes and CNT scaffold-stabilized Zn anodes. **b** Rate performance at various current densities of 0.1–5 mA cm^−2^ for 1 h. **c** SEM image of Zn electrodes in Zn||Zn symmetrical cells. **d** SEM image of Zn electrodes in Zn@CNTs symmetrical cells. **e** SEM image of CNT scaffolds in Zn@CNTs symmetrical cells (after cycling tests). Copyright 2019 Elsevier [91]. **f** SEM images of a ZF anode and **g** SEM images of a ZF@CB-NFC anode. **h** Cycling performances at 0.2 A g^−1^. **i** Coulombic efficiencies as a function of the cycle number. **j** Zn symmetrical cells with ZF and ZF@CB-NFC electrodes: galvanostatic charge/discharge (GCD) curves of 200 cycles at 0.5 mA cm^−2^. **k** Zn symmetrical cells with ZF and ZF@CB-NFC electrodes: voltage profiles of the 1st–10th cycles at 0.5 mA cm^−2^. Copyright 2020 Elsevier [92]

As a widely used one-dimensional material, CNTs are lightweight and have excellent chemical properties. Because unmodified Zn produces dendrites that cause adverse reactions at the Zn anode/electrolyte interface, leading to the failure of RAZIBs (Fig. 5a), Yang et al*.* [91] built self-supporting, highly flexible, and conductive CNT/paper scaffolds to stabilize Zn metal anodes. On the surface of the Zn electrode, the porous skeleton of the scaffold mechanically regulated the deposition position of Zn^2+^, and the conductive CNT network maintained a homogenized electric field. In addition, the assembled Zn@CNT symmetrical cells exhibited a more stable charge/discharge behavior (Fig. 5b). The modified ZF showed no significant changes after cycling (Fig. 5c, d). The CNT scaffolds in symmetrical cells had the same appearance after circulation (Fig. 5e).

Carbon black is also a widely used carbon-based material. Chen et al. [92] modified Zn anodes with C black coatings and nanofibrillar cellulose adhesives. By modifying zinc foil with a C black coating and a nanofibrillating cellulose (NFC) binder, the dendrite growth and side reactions on the anode are eliminated to achieve an excellent interfacial stability between the anode and electrolyte. After 100 cycles, many dendrites grew on the surface of the ZF (Fig. 5f). The anode comprising ZF modified with a C black coating and NFC binder (ZF@CB-NFC) maintained a uniform surface before and after cycling (Fig. 5g). The cell composed of the modified anodes had a better cycling stability and CE (Fig. 5h, i). The symmetrical cell composed of the modified Zn anode exhibited a better cycling performance and lower polarization voltage (Fig. 5j, k).

In summary, the carbon-based material coatings, such as rGO, CNTs, and AC, are conductive protective layers. They have a certain mechanical strength, which effectively inhibits the growth of dendrites and prevents dendrites from penetrating the separator. More importantly, carbon-based materials with high electrical conductivity can provide a strong electric field strength, which effectively avoids the accumulation of charges and provides a uniform interface electric field. Table 1 summarizes the currently reported coating modification strategies for carbon-based materials. However, it should be noted that the service life of a functional conductive protective layer is not sufficiently long, and long-term operation may reduce the battery performance. Therefore, it is necessary to explore more suitable carbon-based materials as interfacial coatings for Zn anodes, which provide a uniform interfacial electric field and an ultra-long service life.

**Table 1:** Summary of recently reported coatings modification strategies for carbon-based and metal-based materials

Anode materials	Voltage hysteresis	Lifespan	References
rGO-coated Zn foil	≈20 mV (1 mA cm^−2^)	300 h (1 mA cm^−2^, 1 mAh cm^−2^)	[69]
rGO-coated Zn foil	≈170 mV (10 mA cm^−2^)	80 h (10 mA cm^−2^, 2 mAh cm^−2^)	[87]
NGO-coated Zn foil	≈32 mV (1 mA cm^−2^)	1200 h (1 mA cm^−2^, 1 mAh cm^−2^)	[74]
AC-coated Zn foil	≈20 mV (1 mA cm^−2^)	200 h (1 mA cm^−2^, 1 mAh cm^−2^)	[90]
CNT scaffold-stabilized Zn anode	≈36 mV (0.1 mA cm^−2^)	1800 h (0.1 mA cm^−2^, 0.5 mAh cm^−2^)	[91]
ZF@CB-NFC anode	160 mV (0.5 mA cm^−2^)	400 h (0.5 mA cm^−2^, 0.5 mAh cm^−2^)	[92]
Zn|In anode	54 mV (0.2 mA cm^−2^)	1500 h (0.2 mA cm^−2^, 0.2 mAh cm^−2^)	[93]
NA-Zn-60 anode	≈80 mV (0.25 mA cm^−2^)	2000 h (0.25 mA cm^−2^, 0.05 mAh cm^−2^)	[94]

### Metal-Based Materials

Some metal-based materials are considered promising coating materials for Zn-ion batteries owing to their Zn affinity and excellent electrical conductivity. Zincophilicity can increase the number of nucleation sites for Zn^2+^, thereby promoting the uniform deposition of Zn^2+^. On the other hand, some metals have a uniform interfacial electric field because of their conductivity, which adsorbs Zn^2+^ and accelerates ion transport. Therefore, metal coating effectively suppresses the formation of large and uneven dendrites/protrusions.

**Fig. 6:**
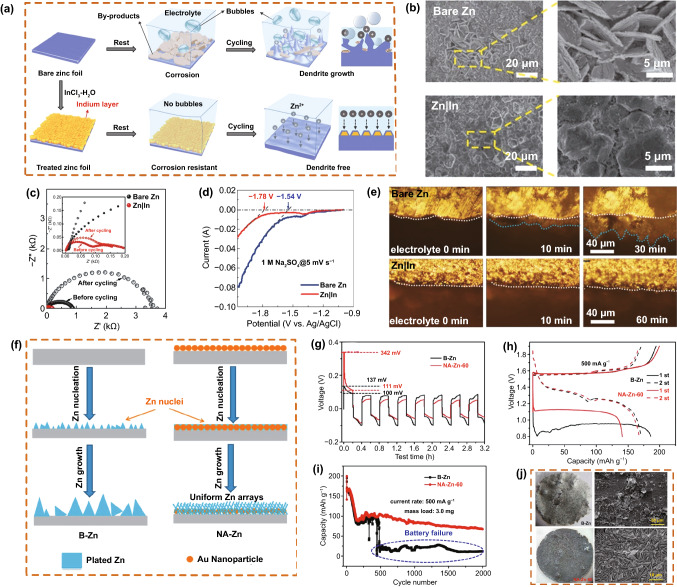
**a** Schematics and characterization of Zn and Zn|In foils. **b** SEM images of bare Zn and Zn|In in symmetrical cells after the first plating process at 1 mA cm^−2^ for 1 mAh cm^−2^. **c** Electrochemical impedance spectroscopy of bare Zn and Zn|In before and after cycling in symmetrical cells with an aqueous ZnSO_4_ electrolyte and **d** Linear sweep voltammetry curves of bare Zn and Zn|In in a 1 M aqueous Na_2_SO_4_ electrolyte at a scan rate of 5 mV s^−1^. **e** Operando optical microscope images of bare Zn and Zn|In in an aqueous ZnSO_4_ electrolyte. Copyright 2020 Wiley-VCH [93]. **f **Schematic illustration of the Zn stripping/plating process on a bare Zn and NA-Zn. **g** Initial discharge/charge profiles of Zn|Zn symmetrical cells at a current density of 0.25 mA cm^−2^. **h** Voltage profiles.** i** Cycling performance of Zn|CNT/MnO_2_ cells at 500 mA g^−1^. **j** Optical and SEM images of bare Zn anode and NA-Zn-60 anode. Copyright 2019 American Chemical Society [94]

Han et al*.* [93] studied metallic In coatings with in situ and ex situ characterization methods. The In metal layer, with the dual function of corrosion inhibitor and nucleation agent, covered the Zn surface, remarkably inhibiting corrosion of the Zn electrode (Fig. 6a). The modified In layer had an inhibitory effect on dendrite growth, making the electrode present a uniform and dense Zn coating after cycling (Fig. 6b). Concurrently, the Zn|In cell exhibited a lower electrochemical impedance (Fig. 6c). The initial potential of the hydrogen evolution reaction was lower, which proves that the In metal coating effectively inhibits the hydrogen evolution reaction (Fig. 6d). Compared with rough plating on the bare Zn surface, the dense galvanized layer on Zn|In exhibited a more uniform surface (Fig. 6e). Cui et al*.* [94] studied a heterogeneous seed method to guide the morphology evolution of plated Zn. Nano-Au particles were used as seed crystals to inhibit the formation of delayed interactions, thereby achieving a stable stripping/plating process (Fig. 6f). A symmetrical battery with the nano-Au-modified Zn anode (NA-Zn) electrode exhibited a more evident overpotential (Fig. 6g). A NA-Zn|CNT/MnO_2_ cell showed excellent performance in the voltage distribution and cycle performance of constant current charge and discharge (Fig. 6h, i). After cycling, the NA-Zn-60 electrode exhibited a relatively uniform surface morphology, as compared to bare ZF (Fig. 6j).

Like carbon-based materials, metal-based materials with high conductivity provide a uniform interfacial electric field while inhibiting dendrite growth. In addition, the metal layer with an affinity for Zn increases the number of nucleation sites for Zn^2+^ and induces uniform Zn deposition. The recently reported coating modification strategies for metal-based materials are summarized in Table 1. However, precious metals are expensive, which significantly hinders the wide application of metal coatings on the surface of Zn anodes. Therefore, it is necessary to explore metal materials with large reserves and low prices as an anode protective layer. The preparation method of the metal protective layer should also be simplified; the above-mentioned chemical substitution reaction is of great significance.

### Non-metallic Inorganic Materials

Inorganic non-metallic materials have the advantages of persistent stability, environmental protection, low cost, and easy availability. They are widely used as Zn anode coating materials. Some inorganic non-metals with a high dielectric constant and low conductivity induce Zn-ion deposition with different working mechanisms to redistribute Zn ions. Thus, the ion concentration gradient generated by the preferred ion flux near the Zn nucleation cluster is reduced to inhibit dendritic growth and side reactions, improving the electrochemical performance of the Zn anode. Various inorganic non-metallic materials, such as metal oxides, kaolin, and salts, have been reported for interface engineering strategies.

**Fig. 7:**
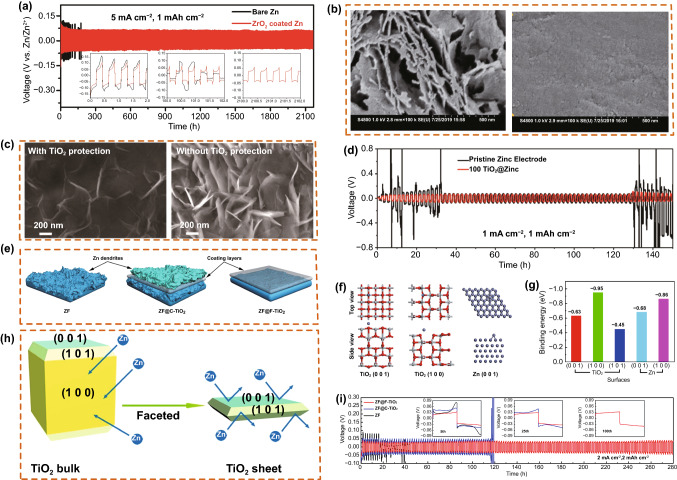
**a** Voltage profiles of metallic Zn stripping/plating in a bare Zn and ZrO_2_-coated Zn symmetrical full-cell at 5 mA cm^−2^ for 1 mAh cm^−2^. **b** Digital images and corresponding SEM images of a bare Zn and ZrO_2_-coated Zn anode after 100 cycles at 5 mA cm^−2^ with 2.5 mAh cm^−2^. Copyright 2020 Royal Society of Chemistry [55].** c** *Ex situ* SEM images of 100TiO_2_@Zn and a pristine Zn anode. **d** Cyclic stripping/plating process of symmetrical cells using 100TiO_2_@Zn and pristine Zn at 1 mA cm^−2^. Copyright 2019 Wiley-VCH [47]. **e** Schematic illustration of the Zn plating process with different coating layers. **f **Calculation models of Zn absorbed on a TiO_2_ (0 0 1) facet, TiO_2_ (1 0 0) facet, and Zn (0 0 1) facet. **g** Calculated binding energies of Zn atom with different facets. **h** Schematic illustration of the interaction between Zn and anatase TiO_2_ with different exposed facets. **i** Cycling performance of Zn||Zn symmetrical cells at 2 mA cm^−2^ for 2 mAh cm^−2^. Copyright 2020 Springer Nature [95]

Metal oxides have been widely studied for inorganic non-metallic coatings in Zn anodes. By employing inorganic non-metallic oxides as Zn anodes, Yi et al. [55] synthesized a nano-ZrO_2_ coating on a Zn anode by a simple sol–gel method, which was beneficial for controlling the nucleation position of Zn^2+^ and achieving the rapid transport of Zn^2+^. The ZrO_2_-coated Zn anode exhibited a longer cycle life and lower polarization (Fig. 7a). After 100 cycles, the ZrO_2_-coated Zn anode presented a flat and uniform morphology under the same conditions as the bare Zn anode (Fig. 7b). Mai et al. [47] used a TiO_2_ coating, deposited by the atomic layer deposition (ALD) technique, as the protective layer of a metal Zn anode. The use of ultra-thin TiO_2_ coating, as a stable passivation of Zn metal, avoided direct contact between the Zn anode and electrolyte. The 100 ALD cycles of TiO_2_ on the Zn plate only deposited a small number of thin slices, as compared to bare Zn (Fig. 7c), and symmetrical cells coated with TiO_2_ on the Zn plate showed excellent cycling stability (Fig. 7d). Wang et al. [95] studied the effects of crystal orientation on the affinity of Zn. As a protective layer on the Zn anode, multi-faceted Ti dioxide has a low affinity for Zn, which limits the formation of dendrites (Fig. 7e). When commercial TiO_2_ is used as the intermediate layer, Zn tends to grow on the surface of TiO_2_ and has a high affinity for Zn. By establishing a model of Zn atoms adsorbed on the surface of TiO_2_, the affinity of the TiO_2_ surface to Zn can be determined (Fig. 7f). As shown in Fig. 7g, Zn is more inclined to deposit on the TiO_2_ surface than on the Zn surface. By controlling the exposure of a specific surface, an appropriate protective material can be obtained (Fig. 7h). Through the cyclic stability test, the ZF@F-TiO_2_/ZF@F-TiO_2_ symmetrical cell was charged and discharged for a long time and had a relatively long life (Fig. 7i).

**Fig. 8:**
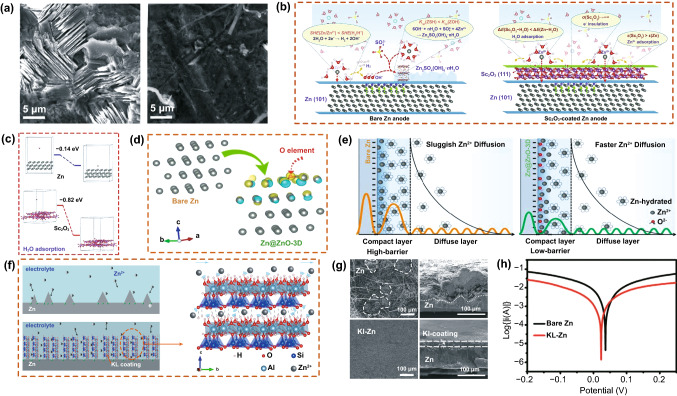
**a** SEM images of bare Zn after cycling. Copyright 2020 Royal Society of Chemistry [96]. **b** Long-term cycling stability of bare Zn and 100Al_2_O_3_@Zn with a 200-µl electrolyte. **c** Schematic diagram of the reaction process on the surface of a bare Zn anode and Sc_2_O_3_-coated Zn anode. Copyright 2020 Elsevier [97]. **d** Differential charge density distribution of Zn@ZnO-3D calculated by first-principle calculations. **e** Electric double-layer structure in the vicinity of the anode and the corresponding energy barrier. Copyright 2016 Royal Society of Chemistry [68]. **f** Schematic diagram of Zn^2+^ deposition on the surface of naked Zn and KL-Zn and detailed schematic diagram of the limited Zn^2+^ transport in kaolin. **g** SEM images of the surface and cross-sectional morphology of the KL-Zn anode after 600 cycles with a MnO_2_ cathode under 0.5 A g^−1^. **h** Linear polarization curves for the corrosion of bare Zn and KL-Zn. Copyright 2020 Wiley-VCH [98]

In addition, Liu et al. [96] prepared Al_2_O_3_ coatings using ALD. By coating ultra-thin Al_2_O_3_ on the surface of a Zn plate, the corrosion resistance was improved and the growth of Zn dendrites was effectively inhibited (Fig. 8a). 100Al_2_O_3_@Zn exhibited good cyclic stability and a small voltage lag during repeated Zn plating and stripping, as compared to bare Zn. Fang et al. [97] prepared a new type of Zn anode with a hydrophobic multi-channel Sc_2_O_3_ coating and realized a layered absorption effect on the anode. As shown in Fig. 8b, the Sc_2_O_3_ coating not only provided a physical barrier to prevent direct contact between the Zn anode and electrolyte, but also formed H bonds with H_2_O molecules. The energy of water absorbed on the Sc_2_O_3_-coated Zn anode was lower than that on the bare Zn anode (Fig. 8c). Zhou et al. [68] developed a 3D nanoporous ZnO structure (Zn@ZnO-3D) by a one-step liquid deposition method to modify the surface of the Zn anode, which adjusted the solvation sheath structure of Zn^2+^ in electric double layers. By comparing the morphologies, the ZnO-3D structure-modified Zn anode inhibited dendrite growth with good cycling stability. Furthermore, a first-principles calculation confirmed that the extra surface charge concentration reduced the energy barrier of Zn^2+^ desolvation and accelerated the kinetics of Zn^2+^ deposition on Zn@ZnO-3D (Fig. 8d, e). In addition, Zhou et al. [98] prepared a kaolin-coated Zn anode (KL-Zn), which provided abundant active adsorption sites for Zn^2+^, and its porous structure greatly assisted in realizing uniform dendrite-free Zn deposition. During the 3D diffusion of KL-Zn, the homogeneous porous structure of kaolin constrained Zn^2+^ and inhibited the formation of Zn dendrites (Fig. 8f). After a full-cell cycle test, the KL-Zn electrode surface retained its morphological stability (Fig. 8g). The KL-Zn anodes exhibited superior corrosion resistance and long-term cycling stability (Fig. 8h).

**Fig. 9:**
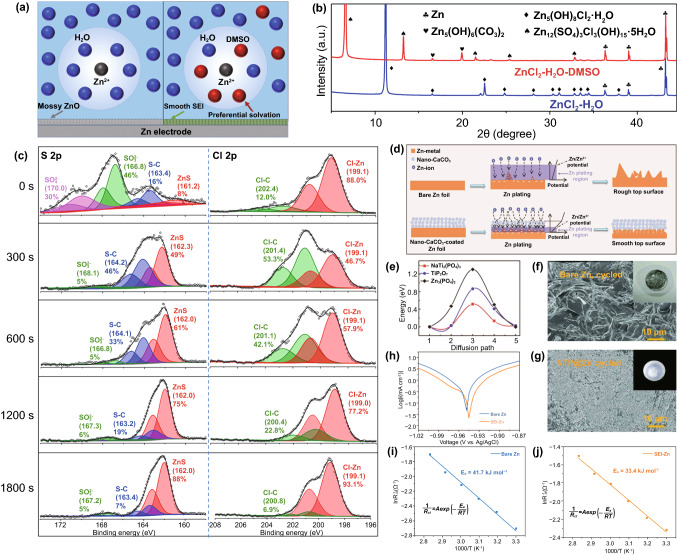
**a** Scheme of the Zn^2+^solvation structure and Zn surface passivation in H_2_O and H_2_O-DMSO solvents. **b** XRD patterns of Zn anodes after plating/stripping cycles in ZnCl_2_-H_2_O-DMSO and ZnCl_2_-H_2_O electrolytes. **c** XPS characterization of the SEI formed on Zn cycled in ZnCl_2_-H_2_O-DMSO electrolyte. Copyright 2020 American Chemical Society [83]. **d** Schematic diagram of the morphological evolution of bare and nano-CaCO_3_-coated ZFs during Zn stripping/plating cycling. Copyright 2018 Wiley-VCH [99]. **e** Proposed migration energy barriers of Zn ions in bulk NTP, TiP_2_O_7_, and Zn_3_(PO_4_)_2_. The SEM images of **f **bare Zn and **g** NTP@Zn foil after 100 stripping/plating cycles. Copyright 2020 Wiley-VCH [100]. **h** Linear polarization curves showing the corrosion on bare Zn and SEI-Zn electrodes. Corresponding Arrhenius curves of Zn symmetric cells at different temperatures: **i** bare Zn and** j** SEI-Zn. Copyright 2021 Wiley-VCH [101]

Coatings on the surface of the Zn anode can be obtained by in situ and ex situ methods. The in situ SEI layer is an important strategy for the preparation of high-performance Zn metal anodes. An in situ SEI layer was constructed on the surface of the Zn anode by adding additives to the electrolyte. Electrolyte additives can change the solvation sheath of Zn ions, and the formed SEI layer can effectively prevent the growth of dendrites and inhibit the decomposition of solvated H_2_O. Wang et al*.* [83] inhibited water reduction and Zn dendrite growth in dilute aqueous electrolytes by adding dimethyl sulfoxide (DMSO) to ZnCl_2_. Moreover, the preferential solvation of DMSO changed the Zn^2+^ sheath of H_2_O solvation (Fig. 9a). The SEI compositions on the Zn anodes were analyzed by X-ray diffraction (XRD) (Fig. 9b) and X-ray photoelectron spectroscopy (XPS) (Fig. 9c), and it was clearly visible in the XPS images that the ZnS content increased gradually with an increase in time. Therefore, the contribution of the DMSO additive to the formation of the Zn_12_(SO_4_)_3_Cl_3_(OH)_15_·5H_2_O-ZnSO_3_-ZnS SEI is closely related to the solvation structure of Zn^2+^ ions in the electrolyte. SEI allows Zn^2+^ transport but prevents H_2_O infiltration, which further inhibits water reduction and Zn dendrite growth.

Some inorganic salts are also suitable coating materials for the protection of Zn anodes. Kang et al. [99] constructed Zn|ZnSO_4_ + MnSO_4_|MnO_2_ aqueous batteries with nano-CaCO_3_-coated Zn metal anodes. During the Zn stripping/plating cycle, the electrical insulation of the CaCO_3_ coating led to the deposition of Zn under the coating, thereby forming a three-layer structure of CaCO_3_/Zn sheet/ZF, which effectively prevented dendrite generation and short-circuit problems (Fig. 9d). After the Zn stripping/plating cycle, the ZF covered by nano-CaCO_3_ formed a compact and uniform micro-sheet layer, enabling a longer life for the Zn metal anode. Zhu et al. [100] hydrothermally synthesized a fast ion conductor, NaTi_2_(PO_4_)_3_ (NTP), on the surface of a Zn anode as a solid electrolyte protective layer. DFT calculations and cyclic voltammetry tests showed that NTP has a higher ionic conductivity than other insoluble phosphates (Fig. 9e). The internal transport/mobility of Zn^2+^ was used in the NTP layer as an "ion can pass through the fence." The NTP layer with a thickness of 20–25 μm not only prevented side reactions and Zn dendrites (Fig. 9f, g), but also improved the reversibility and electrochemical performance of the Zn anode. Mao et al. [101] introduced Zn(H_2_PO_4_)_2_ salt into an aqueous electrolyte to form a solid and highly Zn^2+^-conductive SEI layer (hopeite) in situ by taking advantage of the local pH increase caused by water decomposition. Compared with bare Zn, the corrosion potential of SEI-Zn increased, the tendency of the corrosion reaction was smaller, and the reaction rate decreased, which had a protective effect on the Zn electrode (Fig. 9h). In addition, by measuring the activation energy of the desolvation of symmetrical cells during Zn plating at different temperatures, it was found that the activation energy of SEI-Zn was lower than that of bare Zn, which indicates that the SEI layer provides a rapid transport channel for Zn^2+^ and accelerates the reaction kinetics (Fig. 9i, j).

Briefly, non-metallic inorganic material coatings are non-conductive protective layers, and there are no problems caused by uneven strength in the electric field. This type of inorganic coating uses a porous channel structure to modify the deposition behavior and migration path of Zn^2+^ and induces the orderly deposition of Zn^2+^ along the channels. In addition, the uniform flux of the electrolyte was controlled to induce a smaller Zn nucleation size by the restriction effect. Table 2 presents a summary of recently reported coating modification strategies for non-metallic inorganic materials. However, from the perspective of ion and electron transport, the dense interface layer hinders the migration of ions on the electrode surface. The use of these electrochemically inert protective materials reduces the rate performance of the anode to a certain extent.

**Table 2:** Summary of recently reported coatings modification strategies for non-metallic inorganic materials

Anode materials	Voltage hysteresis	Lifespan	References
Nano-ZrO_2_-coated Zn anode	32 mV (5 mA cm^−2^)	2100 h (5 mA cm^−2^, 1 mAh cm^−2^)	[55]
Ultrathin TiO_2_-coated Zn anode	57 mV (1 mA cm^−2^)	150 h (1 mA cm^−2^, 1 mAh cm^−2^)	[47]
TiO_2_ of low Zn affinity-coated Zn anode	≈40 mV (1 mA cm^−2^)	460 h (1 mA cm^−2^, 1 mAh cm^−2^)	[95]
Al_2_O_3_-coated Zn anode	36.5 mV (1 mA cm^−2^)	500 h (1 mA cm^−2^, 1 mAh cm^−2^)	[96]
Sc_2_O_3_-coated Zn anode	71 mV (1 mA cm^−2^)	200 h (1 mA cm^−2^, 1 mAh cm^−2^)	[97]
Zn@ZnO-3D anode	43 mV (5 mA cm^−2^)	500 h (5 mA cm^−2^, 1.25 mAh cm^−2^)	[68]
Kaolin-coated Zn foil	≈70 mV (4.4 mA cm^−2^)	800 h (4.4 mA cm^−2^, 1.1 mAh cm^−2^)	[98]
Zn anode with ZnS SEI	≈41 mV (0.5 mA cm^−2^)	1000 h (0.5 mA cm^−2^, 0.5 mAh cm^−2^)	[83]
Nanoporous CaCO_3_-coated Zn anode	80 mV (0.25 mA cm^−2^)	836 h (0.25 mA cm^−2^, 0.05 mAh cm^−2^)	[99]
NTP@Zn	50 mV (1 mA cm^−2^)	260 h (1 mA cm^−2^, 1 mAh cm^−2^)	[100]
Zn with hopeite SEI	≈80 mV (1 mA cm^−2^)	1200 h (1 mA cm^−2^, 1 mAh cm^−2^)	[101]

### Polymers

Polymer coatings, such as PA, 502 glue, polyvinyl butyral (PVB), and polyacrylonitrile (PAN), are generally used as artificial SEIs. These polymers normally exhibit good ionic conductivity and abundant polar groups, which interact with solvated metal ions. Additionally, the coatings not only effectively protect Zn from the influence of H_2_O but also manipulate the uniform nucleation sites of Zn plating/stripping.

**Fig. 10:**
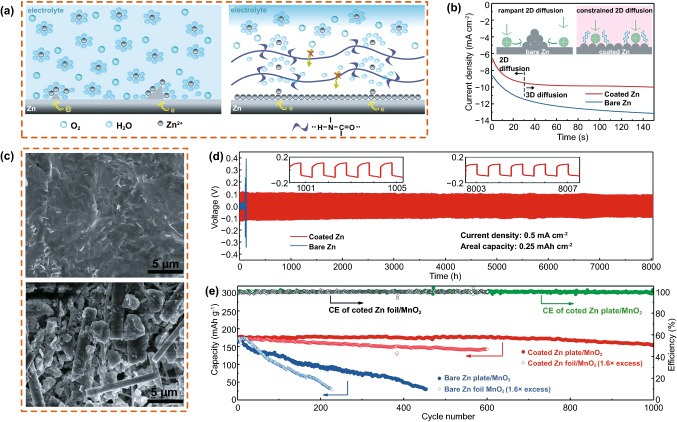
**a** Schematic diagrams for Zn deposition on bare Zn and coated Zn. **b** Chronoamperograms of bare Zn and coated Zn at a -150 mV overpotential. Insets: schematics of the Zn^2+^ diffusion and reduction processes on bare and coated Zn electrodes. **c** SEM images of the surface of a coated Zn plate anode after 1000 cycles and a bare Zn plate after 450 cycles. **d** Long-term galvanostatic cycling of symmetrical Zn cells with coated and bare Zn plates at 0.5 mA cm^−2^. **e** Cycling performance at a current density of 2 C. Copyright 2019 Royal Society of Chemistry [80]

Cui et al. [80] proposed that the PA coating possesses a strong coordination ability for metal ions and a unique H bonding network. The coating reduces the number of harmful water molecules involved in the side reactions. Consequently, the water molecules that are bound by the H bonding network cannot destroy the solvation sheath of Zn^2+^, resulting in a higher number of Zn^2+^ migrations (Fig. 10a). In addition, the PA coating coordinates the uniform migration of Zn^2+^ by densifying the crystal nucleus (Fig. 10b) and acts as a water/O_2_-resistant buffer layer to isolate Zn from the electrolyte, thus inhibiting corrosion and passivation of the Zn anode (Fig. 10c). A symmetrical Zn battery with a polymer-modified Zn anode could work in reverse for 8000 h without Zn dendrites (60 times that of bare Zn) through this synergistic effect (Fig. 10d). The capacity retention rate and CE of the Zn/MnO_2_ cell with coated Zn were 88% and 99%, respectively, after 1000 cycles (Fig. 10e).

**Fig. 11:**
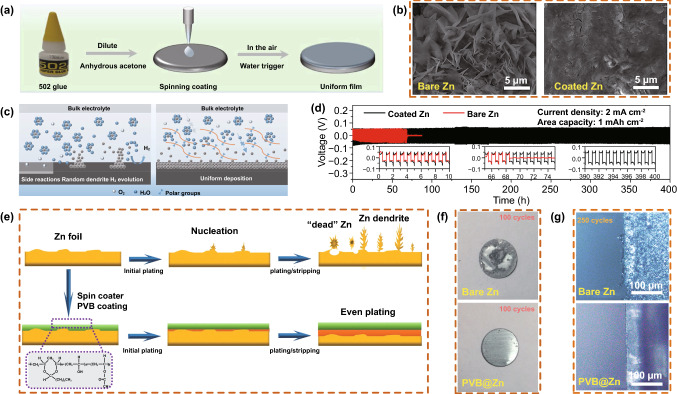
**a** Schematic diagram of the preparation of a 502 glue protective layer. **b** Morphology of bare ZF and 502-decorated ZF obtained from symmetrical Zn cells after Zn stripping/plating for 100 cycles at 0.5 mA cm^−2^ for 0.25 mAh cm^−2^. **c** Schematic diagram of the mechanism of 502 glue for suppressing Zn dendrite. **d** Long-term cycling stability for symmetrical cells with various current densities and capacities. Copyright 2020 Elsevier [102]. **e** Schematic illustration of the morphological evolution for both a bare Zn and PVB@Zn symmetrical cell during repeated stripping/plating cycles.** f** Digital images of Zn and PVB@Zn electrodes that were stripped out of the cells after 100 cycles. **g** Zn electrodes and PVB@Zn electrodes in symmetrical transparent cells, along with the specified numbers of plating/stripping cycles. Copyright 2020 Royal Society of Chemistry [103]

Cao et al. [102] applied 502 glue (solvent-free cyanoacrylate adhesive) uniformly on the Zn surface using the spin-coating method (Fig. 11a). This coating has the important function of acting as an artificial SEI film to effectively inhibit the corrosion of O_2_ and H_2_O molecules on the Zn anode, the formation of Zn dendrites, and the occurrence of other side reactions (Fig. 11b). Additionally, owing to the adsorption effect between Zn^2+^ and the polar cyano groups in the backbones of 502 glue, the Zn nucleation sites are more evenly and orderly distributed (Fig. 11c). Furthermore, the 502 glue-coated Zn cell showed excellent cycling stability and a high CE (99.74%) (Fig. 11d). Hao et al. [103] fabricated a PVB coating with rich polar functional groups. The PVB coating effectively removed the solvated water during Zn plating/stripping and significantly inhibited the occurrence of side reactions (Fig. 11e). The electrolyte on the Zn surface was also evenly distributed by the PVB coating, which guided the dissolution and deposition process of Zn ions. Therefore, employing PVB coatings inhibits the side reactions and growth of Zn dendrites (Fig. 11f, g). Recently, Hu et al. [104] used a simple drop-coating method to prepare a PAN coating on a Zn anode to solve the dendrite problem. Because of the microchannels in the polymer network and the interaction effect between Zn^2+^ and the cyano groups (–CN), the PAN coating combined with Zn salt not only promoted the uniform transport of dissolved Zn^2+^ in the film, but also drove the uniform electrodeposition of Zn metal.

A polymer coating was used as a stable artificial SEI between the electrode and the electrolyte. Polymers have a large number of polar groups and cooperate with Zn^2+^, which provides abundant active sites for nucleation and deposition. In addition, some polymer coatings can reduce the number of harmful water molecules involved in side reactions and effectively inhibit the side reactions, such as hydrogen evolution, corrosion, and passivation. Similar to non-metal inorganic materials, polymers are non-conductive protective layers. The recently reported coating modification strategies for polymers are listed in Table 3. However, these strategies provide an additional quality of inert materials and increase the length of the migration path of Zn^2+^, thereby affecting the rate performance of the anode.

**Table 3:** Summary of recently reported coatings modification strategies for polymer

Anode materials	Voltage hysteresis	Lifespan	References
PA layer/Zn foil	100 mV (0.5 mA cm^−2^)	8000 h (0.5 mA cm^−2^, 0.25 mAh cm^−2^)	[80]
502 glue-coated Zn	≈50 mV (2 mA cm^−2^)	400 h (2 mA cm^−2^, 1 mAh cm^−2^)	[102]
PVB-coated Zn	≈80 mV (0.5 mA cm^−2^)	2200 h (0.5 mA cm^−2^, 0.5 mAh cm^−2^)	[103]
PAN-coated Zn	75 mV (1 mA cm^−2^)	1145 h (1 mA cm^−2^, 1 mAh cm^−2^)	[104]

### Composite Materials

There are various types of Zn anode surface coatings. Moreover, each material has its own advantages and limitations. For example, metals as effective heterogeneous seeds guide the uniform deposition of metallic Zn [94], but are expensive. Most inorganic substances have a porous structure [99], but they are brittle and break easily after long-term cycling or rapid Zn plating/stripping. The organic polymer layer is flexible, but on the one hand, the hydrophobic polymer layer significantly increases the polarization potential of Zn plating/stripping owing to the increase in the nucleation barrier and the limitation of the 2D diffusion of Zn^2+^ [80]. On the other hand, hydrophilic polymers easily dissolve in aqueous electrolytes, thus losing the protection of the Zn anode. Consequently, the preparation of composite coatings is a new strategy for protecting Zn anodes. Current research results show that the metal–organic framework (MOF) has a porous structure and that the organic–inorganic composite coating has a unique organic–inorganic structure. In addition, these composite coatings have good wettability to the electrolyte to suppress water-induced side reactions by the desolvation effect.

**Fig. 12:**
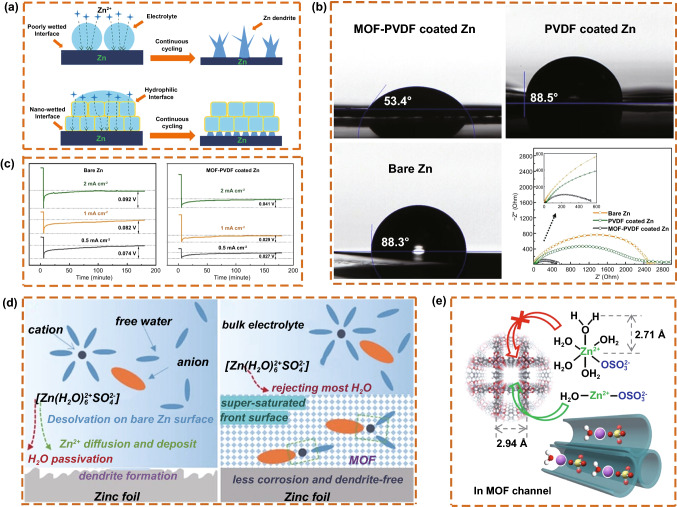
**a** Proposed Zn plating mechanisms on bare Zn and MOF-PVDF-coated Zn. **b** Images of contact angles between the electrolyte and different anodes, and electrochemical impedance spectra of Zn symmetrical cells with different anodes. **c** Voltage curves of Zn plating on bare Zn and coated Zn at various current densities. Copyright 2019 American Chemical Society [105]. **d** Schematic illustration of the Zn surface evolution. **e** Schematic illustration of the highly-coordinated ion complexes of H_2_O-Zn^2+^·OSO_3_^2−^ migrating through MOF channels. Copyright 2020 Wiley-VCH [78]

Pan et al. [105] proposed an artificial composite protective layer composed of a nano-MOF to reconstruct the Zn/electrolyte interface. The MOF particles immersed in the electrolyte formed a good interface contact (Fig. 12a). The microporous structure of the MOF improved the wettability of the Zn anode and created a zincophilic interface, which significantly reduced the interface charge transfer resistance (Fig. 12b). The MOF coating decreased the overpotential of the battery (Fig. 12c), reduced the nucleation energy, and enhanced the zincophilicity of the interface. Qian et al. [78] also studied MOF materials. They used MOF as the front surface layer and MOF channels to maintain a supersaturated electrolyte layer on the Zn anode. Bare Zn suffers from water decomposition and passivation during the desolvation process of Zn-aqueous ionic association [Zn(H_2_O)_6_^2+^SO_4_^2−^]. However, through the mechanism of water inhibition, the MOF layer repelled a large amount of water in advance and obtained the positive side of the super-saturated electrolyte (Fig. 12d, e). Moreover, it is beneficial to guide the uniform deposition of Zn to obtain a dendrite- and corrosion-free Zn anode.

**Fig. 13:**
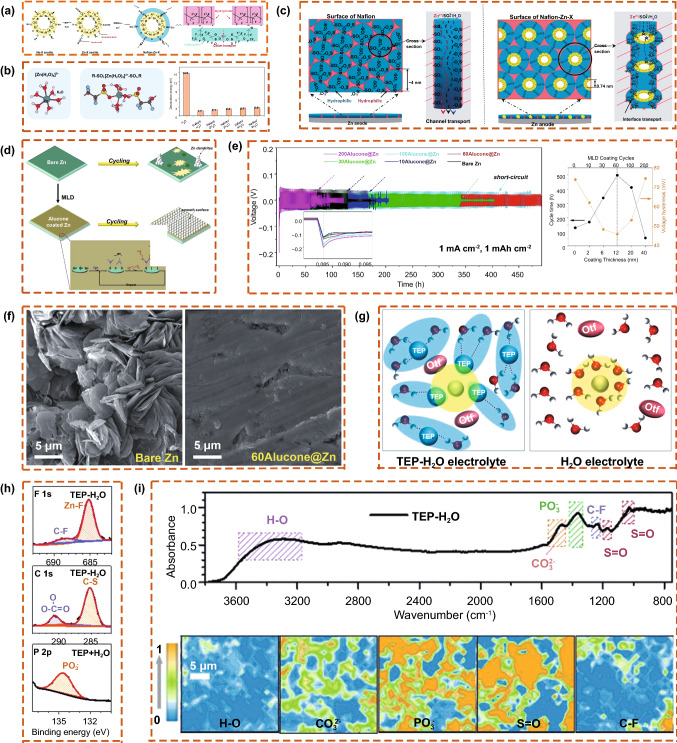
**a** Schematic diagram of the formation process for the ~ SO_3_^−^–Zn^2+^ ~ bridge bond at the interface between organic Nafion and inorganic Zn-X zeolite and the organic structure of Nafion with Zn^2+^. **b** Coordination environment of Zn^2+^ in water and Nafion with 4H_2_O and desolvation energy values of Zn^2+^ in water or in Nafion with various H_2_O. **c** Ion transport mechanisms in Nafion and Nafion-Zn-X protective layers. Copyright 2020 Wiley-VCH [26]. **d** Schematic illustration showing the effect of an inorganic–organic molecular layer deposition (MLD) alucone coating on Zn metal anodes when cycling. **e** Galvanostatic charge–discharge profiles of symmetrical Zn cells with different electrodes at 1 mA cm^−2^ and an areal capacity of 1 mAh cm^−2^, and statistical graphs of cycling time and voltage hysteresis against the coating thickness/MLD cycles. **f** Top-view morphologies of cycled bare Zn and 60Alcuone@Zn. Copyright 2020 Royal Society of Chemistry [106]. **g** Electrolyte solvation structure and its influence on the V_2_O_5_ dissolution of Zn(OTf)_2_-TEP-H_2_O electrolyte and pure aqueous electrolyte. Characterization of the interphase on the cycled Zn surface: **h** XPS fitted curves of the F 1s, C 1s, and P 2p elements and **i** the interference reflection microscopy spectrum of the cycled Zn electrode and the acquired distribution of surface functional groups. Copyright 2021 Wiley-VCH [84]

Yang et al*.* [26] designed and synthesized a type of organic–inorganic composite protective layer (Nafion-Zn-X). They incorporated hydrophilic Zn-X zeolite into the hydrophilic region of the Nafion membrane. In the composite layer, Zn^2+^ connected the interface between Zn-X and Nafion, forming a well-bridged and dense organic–inorganic interface, so that ion migration changed from the Nafion channel migration to the organic–inorganic interface transition mechanism (Fig. 13a). The Nafion-based films exhibited a lower desolvation energy (Fig. 13b), which was beneficial for improving the Zn plating/stripping kinetics and for the growth of dendrites. The Nafion-Zn-X composite coating effectively shielded SO_4_^2−^ anions, freed H_2_O to inhibit side reactions (Fig. 13c), and protected the Zn anode. In addition, Liu et al. [106] synthesized alucone (an inorganic–organic hybrid coating) using a molecular deposition technique. The coating improved the wettability of the Zn anode and led to the uniform deposition of Zn (Fig. 13d). Compared with the bare Zn anode, the coated Zn anode exhibited a long cycle life and low voltage hysteresis (Fig. 13e) and inhibited dendrite growth on the surface of the Zn anode (Fig. 13f). Mao et al. [84] used the flame-retardant triethyl phosphate (TEP) as a co-solvent by adjusting the solvation structure of the non-aqueous electrolyte. TEP preferentially forms a TEP-occupied inner solvation sheath around Zn^2+^ and strong H bonding with H_2_O (Fig. 13g), leading to a robust polymeric-inorganic interphase (poly-ZnP_2_O_6_ and ZnF_2_) (Fig. 13h, i) on the Zn anode, effectively preventing dendrite growth and the side reactions of H_2_O.

Table 4 presents a summary of recently reported coating modification strategies for composite materials. Composite coatings are prepared by combining different materials to overcome the limitations of a single material. Composite material coatings are difficult to prepare and are not easily mass produced. In contrast, carbon-based materials are easily available and have a significant effect on dendrite suppression. Metal-based materials with high conductivity are optional coating materials, but precious metals are expensive, which hinders their wide application. Non-metal inorganic materials and polymers are easy to prepare and inexpensive. However, the use of these electrochemically inert protective materials reduces the rate performance of the anode to a certain extent. Therefore, it is necessary to continuously explore easily prepared, low-cost, and excellent performance coating materials as the anode protective layer in the future.

**Table 4:** Summary of recently reported coatings modification strategies for composite materials

Anode materials	Voltage hysteresis	Lifespan	References
MOF-PVDF coated Zn	≈150 mV (3 mA cm^−2^)	500 h (3 mA cm^−2^, 0.5 mAh cm^−2^)	[105]
MOF-coated Zn foils	≈85 mV (0.5 mA cm^−2^)	3000 h (0.5 mA cm^−2^, 0.5 mAh cm^−2^)	[78]
Zn@Nafion-Zn-X composite anode	≈50 mV (2 mA cm^−2^)	1000 h (2 mA cm^−2^, 0.5 mAh cm^−2^)	[26]
Alucone-coated Zn	≈103 mV (3 mA cm^−2^)	780 h (3 mA cm^−2^, 1 mAh cm^−2^)	[106]
Zn with poly-ZnP_2_O_6_ and ZnF_2_ interphase	150 mV (1 mA cm^−2^)	1500 h (1 mA cm^−2^, 1 mAh cm^−2^)	[84]

## Interface Modification of Electrolyte/Zn Anode by Electrolyte Additives

In addition to surface coatings, electrolyte additives are an important strategy in interface engineering. Electrolyte additives are used to adjust the surface morphology of the Zn anode and inhibit side reactions, such as Zn dendrite growth, hydrogen evolution reaction, and Zn surface corrosion and passivation. Various electrolyte additives have been applied to RAZIBs to protect the Zn anode by electrostatic shielding, crystallographic orientation induction, and modulation of the coordination status mechanism. According to previous research results, electrolyte additives can be divided into ionic and non-ionic additives.

### Ionic Additives

Ionic additives regulate the deposition position of Zn^2+^ and inhibit dendrite growth through electrostatic shielding, crystallographic orientation induction, or modulation of the coordination status mechanism to obtain a uniform Zn deposition layer.

**Fig. 14:**
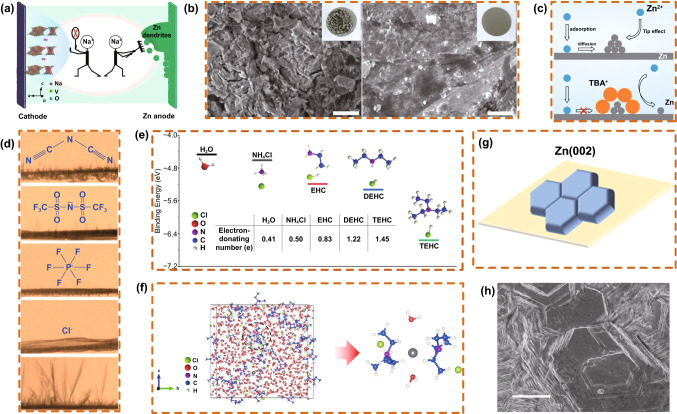
**a** Schematic diagram of a Na_2_SO_4_ additive suppressing the dissolution of NVO nanobelts and the formation of Zn dendrites. **b** SEM images of Zn negative electrodes (1 A g^−1^, 100th cycle) from Zn/NVO cells. Copyright 2020 Springer Nature [70]. **c** Schematics of the Zn^2+^ ion diffusion and reduction processes on electrodes in ZnSO_4_ and a ZnSO_4_ electrolyte with 0.05 mM TBA_2_SO_4_. Copyright 2020 American Chemical Society [107].** d** Real-time X-ray images of Zn deposits at -1.45 V in the presence of additive-free, EMI-Cl, EMI-PF6, EMI-TFSA, and EMI-DCA. Copyright 2016 American Chemical Society [109]. **e** The binding energy between Zn^2+^ and the ligand molecule calculated by DFT. **f **3D snapshot of the molecular dynamics simulation and solvation structure of Zn^2+^ at 3–1 0.5 M TEHC. Copyright 2021 Wiley-VCH [110]. **g** Scheme depicting the proposed columnar structure formation of the deposited Zn on the substrate by Zn(OTf)_2_. **h** SEM image showing the hexagonal-like plate planar morphology of the Zn film grown by Zn(OTf)_2_. Copyright Wiley-VCH [73]

Using positive ions with a lower reduction potential than Zn^2+^ (such as Na^+^) inhibits the growth of Zn dendrites by the electrostatic shielding mechanism. Niu et al. [70] proposed an effective strategy by adding Na_2_SO_4_ to a ZnSO_4_ electrolyte to alleviate the growth of Zn dendrites (Fig. 14a, b). The addition of Na^+^ to the electrolyte could change the dissolution equilibrium of Na^+^ in a NaV_3_O_8_·1.5H_2_O (NVO) cathode, hindering the dissolution of the NVO cathode. The discharge capacity of a cell with Na_2_SO_4_ electrolyte was maintained at 221 mAh g^−1^ with a retention rate of 90% after 100 cycles at 1 A g^−1^. The cell showed a capacity retention rate of 82% after 1000 cycles at a high current density of 4 A g^−1^. Furthermore, using cationic surfactant-type electrolyte additives can have similar effects on the Zn anode. Zhu et al*.* [107] added tetrabutylammonium sulfate (TBA_2_SO_4_) to the electrolyte to form a TBA^+^ protective layer (Fig. 14c) around the Zn dendrites by electrostatic adsorption, which uniformly deposited Zn^2+^ and inhibited the growth of Zn dendrites. Ma et al. [108] studied the influence of adding 1-ethyl-3-methylimidazolium ethyl sulfate to an electrolyte to inhibit the growth of Zn dendrites. The added imidazole ions could be adsorbed on the electrode surface to prevent the growth of Zn dendrites and cooperated with metal ions to form reducing substances to optimize the electrode/electrolyte interface behavior. Furthermore, Hu et al. [109] reported imidazolium ionic additives with different anions (Cl^−^, PF6^−^, TFSA^−^, and DCA^−^) to inhibit the formation of Zn dendrites by increasing the degree of the nucleation overpotential and polarization (Fig. 14d).

Recently, Yang et al*.* [110] proposed the introduction of a series of low-cost "green" molecules with a cation coordination ability into aqueous (ZnCl_2_ and ZnSO_4_) electrolytes. Triethylamine hydrochloride (TEHC) delivered the lowest binding energy of − 6.56 eV according to DFT simulations, indicating that the binding between TEHC and Zn^2+^ was the most stable. Meanwhile, TEHC exhibited the most robust electron-donating capability (Fig. 14e). Small Lewis base molecules, featuring amine or phosphorous groups, compete with excess H_2_O molecules to bind with Zn cations (Fig. 14f). By introducing ligand molecules, the solvation structure of Zn^2+^ can be well adjusted, and the formation of by-products in the water electrolyte can be effectively inhibited, realizing a highly reversible Zn anode.

In addition, manipulating the crystalline direction of Zn deposition can effectively solve the problem of Zn dendrites. Yuan et al. [73] constructed a Zn (002) structure by sulfonate-based electrolyte electrodeposition, which reshaped the Zn coordination in the salt-in-water regime and further at the deposition interface. According to the co-collimation of the Zn (002) plane normal and substrate normal, a hexagonal columnar structure formed by Zn(OTf)_2_ deposited on the substrate was established (Fig. 14g, h). Highly reversible Zn stripping/plating was achieved, and a Zn (002) texture-based aqueous Zn battery with excellent cycling stability was constructed.

In summary, the use of ionic additives results in a more stable electrolyte/Zn anode interface. In future studies, ionic additives that inhibit cathodic dissolution and form a shielding or protective layer on the Zn anode surface are preferred.

### Non-ionic Additives

Non-ionic additives, such as organics, regulate Zn^2+^ deposition and hinder Zn dendrite growth by forming an electrostatic shielding layer on the Zn anode surface. In addition, some organic molecules can adjust the solvation structure of Zn^2+^, inhibit side reactions due to the decomposition of active H_2_O, and prevent dendrite growth.

**Fig. 15:**
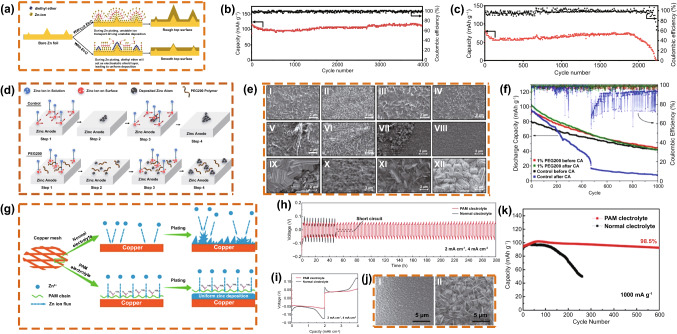
**a** Schematic diagram of the morphological evolution for Zn anodes in a mild aqueous electrolyte with and without Et_2_O additive during Zn stripping/plating cycling. Long-term cycling performance of Zn-MnO_2_ cell at 5 A g^−1^ **b** with Et_2_O additive and **c** without Et_2_O additive. Copyright 2019 Elsevier [71]. **d** Schematic diagram of the step-by-step Zn reduction and deposition process in control and PEG200 electrolytes under negative potential bias on the Zn electrode. **e** SEM image of Zn electrodes after exposure to 1 h CA at -135 mV overpotential vs. open-circuit voltage (OCV) under different conditions. **f** Cycling performance of a small rechargeable hybrid aqueous battery under 4 C before and after exposure to CA for 3 h at -135 mV overpotential vs. OCV. Copyright 2018 Wiley-VCH [114]. **g** Schematic illustration of Zn deposition on a Cu mesh in normal and PAM-added electrolytes. **h** Cycling performance in symmetrical cells and** i** the corresponding voltage profiles in the 10th cycle at 2 mA cm^−2^ for 4 mAh cm^−2^. **j** SEM images of a 3D Zn anode using (I) PAM electrolyte and (II) normal electrolyte after 10 cycles. **k** Cycling performance comparison of the Zn-MnO_2_ full cells using a 3D dendrite-free Zn anode and different electrolytes at 1000 mA g^−1^. Copyright 2019 Wiley-VCH [115]

Xu et al*.* [71] added a small amount of diethyl ether (Et_2_O) to the electrolyte, which greatly improved the electrochemical performance of the Zn-MnO_2_ cells. As shown in Fig. 15a, during the deposition process, the first deposited Zn^2+^ formed a tip with a higher electric field strength, thereby inducing fast ion deposition. The Et_2_O molecules with high polarization properties preferentially adsorbed on the tips of the high-potential Zn dendrites to act as an electrostatic shield. The adsorption of an appropriate amount of Et_2_O molecules repelled the deposition of Zn^2+^ at the tip of the dendrite and promoted deposition in other flat areas to reduce the growth rate of Zn dendrites. In Fig. 15b, c, the reversible capacity of the Zn-MnO_2_ cell and capacity retention were 113 mAh g^−1^ and 97.7% after 400 cycles at 5 A g^−1^, respectively. However, the cell without Et_2_O only had a capacity of 71.8 mAh g^−1^ and failed after 1950 cycles. In addition, Hou et al. [111] found that adding sodium dodecyl sulfate to the electrolyte inhibited the corrosion of the Zn anode and growth of Zn dendrites. Recently, Chao et al*.* [112] added glucose to a ZnSO_4_ aqueous solution to simultaneously modulate the solvation structure of the Zn^2+^ and Zn anode–electrolyte interface. Experiments and theoretical simulations confirmed that glucose can enter the main solvation layer of Zn^2+^, reducing the number of active H_2_O molecules, thereby inhibiting the formation of by-products. Concurrently, glucose molecules tend to be adsorbed on the surface of the Zn anode, inhibiting the random growth of Zn dendrites. Interestingly, Guo et al. [113] reported a practical and low-cost antisolvent strategy. By adding methanol to the ZnSO_4_ electrolyte, the free water and coordination water in the Zn^2+^ solvation sheath gradually interacted with the antisolvent, thereby minimizing water activity and weakening Zn^2+^ solvation.

Mitha et al*.* [114] added polyethylene glycol to an electrolyte to inhibit the growth of Zn dendrites. The addition of 0.1 vol% 200 g mol^−1^ polyethylene glycol (PEG200) electrolyte significantly enhanced the battery performance, and the corresponding mechanism diagram is shown in Fig. 15d. In cells without additives in the electrolyte, Zn^2+^ diffuses in 2D and undergoes aggregation deposition. To minimize the surface energy, Zn^2+^ adsorbs and reduces on the first deposited Zn^2+^ surface, forming large dendrites. When PEG200 is added to the electrolyte, PEG200 adsorbs on the surface of the Zn anode faster than Zn^2+^ and occupies active adsorption sites. PEG200 can act as a physical barrier that limits the deposition of Zn^2+^ at the initial adsorption site. This is conducive to increasing nucleation sites and the formation of significantly denser small dendrites, thereby preventing the uncontrolled growth of large dendrites, which cause short circuits in the cell. The scanning electron microscopy (SEM) image (Fig. 15e) shows the inhibition effect of PEG200 on dendrite formation. As depicted in Fig. 15eI, II, the dendrites of the control sample are larger than those of the PEG200 sample and exhibit a flaky shape. With the cycling, the dendrites of the control sample exhibited a dendritic shape (Fig. 15eIII), "moss-like" shape (Fig. 15eV), cobble-like shape (Fig. 15eVII), boulder-like shape (Fig. 15eIX), and finally returned to a flaky shape (Fig. 15eXI). In the PEG200 sample, densely packed flake dendrites were transformed into evenly and densely packed boulder-shaped dendritic micro-dendrites because of uniform nucleation (Fig. 15eIV, VI, VIII, X, and XII). The addition of PEG200 also contributed to improving the cycle performance of the cell. As shown in Fig. 15f, the cell capacity with the control electrolyte dropped rapidly after the Zn anode was tested by chronoamperometry (CA). In contrast, regardless of whether the Zn anode was subjected to CA, the discharge capacity of the PEG200 sample remained constant after 1000 cycles, which indicates that the PEG200 additive can inhibit dendrites.

Zhang et al. [115] constructed a dendrite-free Zn anode by adding polyacrylamide (PAM) to the electrolyte (Fig. 15g). After adding PAM to the electrolyte, PAM preferentially adsorbed on the surface of a Cu mesh. Zn^2+^ was adsorbed on the acyl groups of PAM and transferred along the polymer chain evenly distributed on the electrode surface. Therefore, the PAM additive increased the number of nucleation sites and guided the uniform deposition of Zn, which is beneficial for forming a flat electroplating surface. The addition of PAM also improved the electrochemical performance of the RAZIBs. The symmetrical cell cycled in a PAM electrolyte for 280 h without short circuit and exhibited a low voltage hysteresis of 93.1 mV (Fig. 15h, i). The cell using the normal electrolyte quickly failed after 52 h of cycling (Fig. 15h, i). In the normal electrolyte, the anode after cycling exhibited many dendrites deposited by thin sheets, and the Zn anode using the PAM electrolyte exhibited no dendrites (Fig. 15j). The PAM electrolyte showed a long life and high capacity retention of 98.5% in the Zn–MnO_2_ full cell (Fig. 15k).

Recently, Zhang et al*.* [116] selected GO powder as an electrolyte additive for Zn anodes and dispersed it in small amounts in the ZnSO_4_ electrolyte to obtain a highly stable and dendrite-free Zn anode. GO electrolyte additives can promote the uniform distribution of the electric field at the interface of the Zn anode, thus improving the binding energy of GO and Zn^2+^, such that Zn^2+^ exhibits an excellent plating/stripping behavior and Zn is uniformly deposited. In addition, the GO electrolyte additive reduced the nucleation overpotential and charge transfer resistance of Zn^2+^, thus providing more nucleation sites and promoting the reaction kinetics. Therefore, compared with the pure ZnSO_4_ electrolyte, the Zn||Zn cell, which used GO as the Zn electrolyte additive, exhibited a service life which exceeded 650 h at 1 mA cm^−2^. Even at 10 mA cm^−2^, its cycle life achieved 140 h. A Zn||Ti cell exhibited an average CE of 99.16% after 100 cycles. A full cell composed of MnO_2_ and GO as the cathode and electrolyte additive, respectively, achieved 250 cycles at 5 A g^−1^, and the capacity retention rate was 93%. The performance improved after the addition of the electrolyte additive.

In summary, the addition of organic matter to the electrolyte can significantly inhibit dendritic growth. Polar molecules preferentially reach initial protrusions to prevent Zn^2+^ deposition by the electrostatic shielding effect. In addition to Et_2_O, other polar molecules, such as ethanol and acetone, can be used as additives. The addition of some organic molecules to the electrolyte can adjust the solvation structure of Zn^2+^, which further demonstrates that adding additives to the electrolyte is an effective way to eliminate dendrites.

## Conclusion and Perspectives

RAZIBs exhibit great application prospects in green energy storage systems owing to their advantages, including low cost, high safety, good durability, and relatively high energy density. Research on cathode materials for RAZIBs has reached a relatively mature stage. However, the poor reversibility of the Zn anode arising from Zn dendrites and side reactions restricts the development of RAZIBs. Interfacial engineering strategies, including surface modification and the addition of electrolyte additives, have received much attention because of their easy operation, diversity of chosen materials, and high efficiency. Based on the working principle of the anode and the expected high performance, the ideal interfacial layer should meet the following requirements: (a) good adhesive force with ZF, maintaining continuous protection of the surface coating; (b) high ionic conductivity and low electrical conductivity, assuring that Zn^2+^ will be deposited on the surface of the Zn anode rather than on the interfacial layer; (c) insolubility and chemical inactivity, inhibiting direct contact between the Zn anode and electrolyte; (d) excellent mechanical properties, accommodating the volume change of the Zn anode during cycling; and (e) moderate thickness, providing an appropriate physical barrier. Research on electrolyte additives is still in its infancy. The influence of additives on the electrolyte structure and many reaction mechanisms are still ambiguous; thus, it is necessary to study the action mechanism of the electrolyte additive to further improve battery performance. Although much progress has been achieved in improving the performance of Zn anodes by interfacial engineering strategies, there is still much space for enhancing the Zn anode performance of RAZIBs.

1. *Development of novel coating materials and methods* Novel coating materials, specifically polymer materials, need to be developed because of the characteristics of uniform active sites, poor conductivity, and good hydrophilicity. Additionally, after considering the interaction of various coatings and chemical bonds, multi-component coating materials deserve further exploration owing to the synergistic effect between their components. For example, a double-layer coating consisting of an electronic conductor layer and an ionic conductor effectively suppresses Zn dendrites and side reactions. The electronic conductor layer on the Zn anode surface provides a more uniform electric field, whereas the ionic conductor realizes the confinement effect of Zn ions. In addition, the binding force, uniformity, and thickness of the coating layers should be comprehensively considered.
2. *Balancing the trade-offs of various aspects* For example, the balance between reaction thermodynamics and ion migration kinetic energy, that is, the inhibition of interface side reactions and ion conduction performance, must be weighed. Moreover, the balance between the energy density and coating thickness should be considered. Based on ensuring the integrity and uniformity of the coating, a thicker coating will cause a greater transmission distance and transmission resistance of Zn ions, which will damage the battery performance. Therefore, it is necessary to appropriately reduce the thickness of the interface layer to improve the quality and volume energy density of the battery system.
3. *Exploring new electrolyte additives* Because the electrolyte contacts both the cathode and the anode, new electrolyte additives can be explored to not only regulate the deposition behavior of Zn ions on the Zn anode surface, but also act on the cathode interface to improve the cycling stability of the cathode material. Furthermore, it is a good strategy to develop super-hydrophilic electrolyte additives, which can effectively fix the free active water molecules in the electrolyte and significantly suppress the side reactions caused by water.
4. *Combining the multiple effects of electrolyte additives* Electrolyte additives can effectively regulate the electrochemical behavior of Zn ions at the Zn anode–electrolyte interface. In addition, the dendrites and side reactions generated on the surface of the metal Zn anode affect each other. However, the function of a single electrolyte additive is relatively limited. Therefore, under the premise of ensuring that there will be no interaction between multiple additives, it is possible to consider adding multiple additives to obtain the best performance.
5. *Nanothickness interface alloying* Nanothickness interface alloying is another effective strategy for achieving high-performance Zn anodes. The current relatively mature metal anticorrosion strategies and electrolyte control methods can be used to alloy Zn anodes. In addition, in view of the interfacial characteristics of the metal itself, synergy regulation with the electrolyte is expected to develop into a novel Zn metal interface engineering strategy.
